# Alterations of Sphingolipid Metabolism in Different Types of Polycystic Ovary Syndrome

**DOI:** 10.1038/s41598-019-38944-6

**Published:** 2019-03-01

**Authors:** Juan Li, Li-Min Xie, Jin-Long Song, Lee-Fong Yau, Jia-Ning Mi, Chun-Ren Zhang, Wan-Ting Wu, Mao-Hua Lai, Zhi-Hong Jiang, Jing-Rong Wang, Hong-Xia Ma

**Affiliations:** 1 Institute of Integrated Traditional Chinese Medicine and Western Medicine, Guangzhou Medical University, Guangzhou, China; 2 Department of Traditional Chinese Medicine, the First Affiliated Hospital of Guangzhou Medical University, Guangzhou, China; 3 State Key Laboratory of Quality Research in Chinese Medicine, Macau Institute for Applied Research in Medicine and Health, Macau University of Science and Technology, Taipa, Macau, China; 4 Department of Clinical Laboratory, the First Affiliated Hospital of Guangzhou Medical University, Guangzhou, China

## Abstract

The roles of sphingolipids in polycystic ovary syndrome (PCOS) are still unknown. This study aimed to investigate the sphingolipid characteristics for different types of PCOS using liquid chromatography-mass spectrometry (LC-MS). A total of 107 women with PCOS and 37 healthy women as normal controls were studied. PCOS patients were further classified into non-obesity with insulin resistance (IR) (NOIR), obesity with IR (OIR), and non-obesity and non-IR (NIR) subgroups. A total of 87 serum sphingolipids, including 9 sphingosines, 3 sphinganines, 1 sphingosine-1-phosphate (S1P), 19 ceramides (Cers), 1 ceramide-1-phosphate, 44 sphingomyelins (SMs), 4 hexosylceramides, and 6 lactosylceramides (LacCers) were analyzed using an improved sphingolipidomic approach based on LC-MS. Notable elevations in the levels of S1P, Cer, and SM were observed in PCOS patients when compared with healthy women, and SM species with long saturated acyl chains showed potential as novel biomarkers of PCOS. In addition, the level of LacCer was only elevated in NIR, and there was almost no change in NOIR and OIR. This study is the first to report the comprehensive sphingolipidomic profiling of different subgroups of PCOS with or without IR or obesity and suggests that serum sphingolipids might be useful as diagnostic biomarkers for different types of PCOS.

## Introduction

Polycystic ovary syndrome (PCOS) is one of the most common endocrine and metabolic disorders in women of reproductive age, and it accounts for 50–70% of anovulatory infertility cases and affects about 5.6% of 19–45-year-old women in China^1,2^. The clinical manifestations are diverse and include oligomenorrhea/amenorrhea, hirsutism, acne, obesity, polycystic ovaries, and infertility^2,3^. PCOS is associated with insulin resistance (IR)/hyperinsulinemia, impaired glucose tolerance, and dyslipidemia^4–7^, and long-term complications include metabolic syndrome, endometrial and breast cancer, and other gynecological tumors^8^.

The etiology of PCOS remains unclear^9^. Pathophysiological changes include hypothalamus-pituitary-ovarian axis dysfunction, hyperandrogenism, hyperinsulinemia, insulin resistance (IR), endocrine dysfunction, adrenal dysfunction, etc.^10–12^. IR is central to the pathogenesis of PCOS, while glucose and lipid disorders are considered to be the general pathology^13^. Compensatory IR is present in about 40% of women with PCOS^14^ and mainly affects classical target tissues of insulin action – such as skeletal muscle, adipose, and liver – and induces systemic glucose and lipid metabolism disorders. IR is a physiological condition in which the target organs become resistant to the effects of insulin. That is, the normal response to a given amount of insulin is reduced, thus weakening insulin’s ability to regulate glucose uptake and glucose utilization^15^.

Several serum/plasma metabolomic studies of PCOS have been carried out using advanced analytical methods, such as liquid or gas chromatography/time-of-flight mass spectrometry (LC or GC/TOF-MS) or nuclear magnetic resonance^16–19^, and these studies have shown that changes in amino acid metabolism, the tricarboxylic acid cycle, and gut microflora, as well as mild perturbations in lipid and glucose metabolism, are correlated with PCOS^20^. A lipidomic analysis of serum/plasma samples from PCOS patients was also recently performed, and disturbances in fatty acid, glycerolipid, and glycerophospholipid metabolism were shown to be related to the pathogenesis of IR in PCOS^21^. In addition, the levels of very long-chain monounsaturated fatty acids were found to be reduced in PCOS patients compared with healthy subjects^22^. However, there has been a lack of studies focusing on sphingolipid alterations in PCOS.

Sphingolipids are a complex family of compounds that are found in eukaryotes as well as some prokaryotes and viruses, and they comprise a small but vital fraction (2–20%) of the membrane lipids^23^. They are involved in a variety of biological processes in eukaryotic cells^24,25^, such as cell proliferation, differentiation, apoptosis, and migration; membrane trafficking; cell-cell interactions; and cell morphology, as well as both intracellular and extracellular signaling^26,27^. However, previous studies on sphingolipids have found that the changes in the levels of sphingolipids in the body directly affect the intensity of insulin signaling^28^. Ceramide (Cer), ganglioside monosialo 3 (GM3), and sphingosine-1-phosphate (S1P) have significant regulatory effects on insulin signaling, and studies have shown that Cer and GM3 play negative roles in insulin signaling and promote the development of IR^29,30^. Conversely, S1P can enhance insulin signaling and inhibit the development of IR^31^. Abnormal lipid metabolism is closely related to the development of IR, and oxidative damage caused by excessive lipid accumulation can induce IR^32^, while sphingolipids mediate the development of IR by regulating insulin signaling molecules^33^. The analysis of whole-serum sphingolipids has been shown to facilitate the understanding of potential mechanisms of some diseases occurrence and development^34^, but the characteristics of sphingolipids in PCOS are unknown.

In the present study, we explored the potential of sphingolipids as biomarkers for PCOS using an integrated sphingolipid analytical platform that combined the advantages of ultra-high performance liquid chromatography (UHPLC)-quadrupole time-of-flight mass spectrometry and UHPLC coupled with triple quadrupole mass spectrometry (UHPLC-QQQ-MS) to qualitatively and quantitatively analyze the sphingolipids in serum samples from healthy women and from different subgroups of PCOS patients.

## Results

### Baseline characteristics

A total of 107 PCOS patients and 37 healthy women were enrolled. Thirty-four (31.8%) of the 107 PCOS patients presented with a body mass index (BMI) < 25 kg/m^2^ and homeostatic model assessment of insulin resistance (HOMA-IR) ≥ 2.14 (the non-obesity with IR (NOIR) group), 41 patients (38.3%) had BMI ≥ 25 kg/m^2^ and HOMA-IR ≥ 2.14 kg/m^2^ (the obesity with IR (OIR) group), and 32 patients (29.9%) had BMI < 25 kg/m^2^ and HOMA-IR < 2.14 kg/m^2^ (the non-obesity and non-IR (NIR) group). The demographic, endocrine, and glycolipid metabolic features of controls and patients with different types of PCOS are described in Table 1. There were no significant differences in age for any of the groups. PCOS patients in the OIR group had a significantly greater BMI, waist-to-hip ratio (WHR), and HOMA-IR; higher levels of total cholesterol (CHOL), triglycerides (TG), and low-density lipoprotein (LDL); and lower levels of high-density lipoprotein (HDL) compared to controls. Except for BMI and HDL, these characteristics were also significantly increased in the NOIR group compared with the controls. However, PCOS in the NIR group only presented with obviously increased luteinizing hormone (LH) to follicle-stimulating hormone (FSH) ratio, LH level, and total testosterone (TT) level compared with controls. Overall, these changes in PCOS patients were clinically related to PCOS type and complemented the metabolomics analysis.

**Table 1:** Baseline characteristics of the three polycystic ovary syndrome (PCOS) subgroups and control subjects.

Characteristic	PCOS			Controls
	NOIR	NIR	OIR	
Number	34	32	41	37
Age (years)	27.88 (26.30–29.46)	28.05 (26.96–29.14)	26.97 (25.56–28.38)	28.11 (26.33–29.89)
BMI (kg/m^2^)	21.03 (20.51–21.56)	20.06 (19.55–20.56)	28.05 (27.02–29.07)*	20.98 (20.15–21.81)
WHR	0.83 (0.81–0.85)*	0.80 (0.78–0.81)	0.88 (0.86–0.90)*	0.79 (0.78–0.80)
HOMA-IR	3.25 (3.01–3.48)*	1.19 (1.10–1.29)	4.85 (3.99–5.71)*	1.04 (0.95–1.14)
CHOL (mg/dl)	5.00 (4.74–5.25)*	4.38 (4.10–4.67)	5.28 (4.90–5.66)*	4.38 (4.17–4.58)
TG (mg/dl)	1.30 (1.10–1.49)*	0.74 (0.68–0.81)	2.08 (1.39–2.77)*	0.78 (0.68–0.88)
HDL (mg/dl)	1.33 (1.23–1.42)	1.51 (1.40–1.61)	1.19 (1.12–1.27)*	1.43 (1.34–1.51)
LDL (mg/dl)	3.18 (2.96–3.39)*	2.61 (2.45–2.77)	3.34 (3.08–3.60)*	2.59 (2.44–2.73)
LH (mIU/ml)	9.61 (7.78–11.44)*	9.83 (7.66–12.00)*	8.71 (7.42–10.00)*	4.63 (4.08–5.19)
LH/FSH	1.60 (1.32–1.89)*	1.41 (1.10–1.71)*	1.56 (1.34–1.78)*	0.67 (0.58–0.76)
TT (ng/ml)	0.64 (0.55–0.72)*	0.60 (0.53–0.67)*	0.64 (0.57–0.72)*	0.42 (0.37–0.47)

Notes: Data are presented as the mean and (95% confidence interval). Differences in continuous variables among groups were analyzed by ANOVA or Kruskal–Wallis test. *p < 0.05.

Abbreviations: NOIR, non-obesity with insulin resistance; OIR, obesity with insulin resistance; NIR, non-obesity and non-insulin resistance; BMI, body mass index; WHR, waist-to-hip ratio; HOMA-IR, homeostasis model assessment of insulin resistance; CHOL, total cholesterol; TG, triglycerides; HDL, high-density lipoprotein; LDL, low-density lipoprotein; LH, luteinizing hormone; FSH, follicle-stimulating hormone; TT, total testosterone.

### Identification of serum sphingolipids

**Figure 1:**
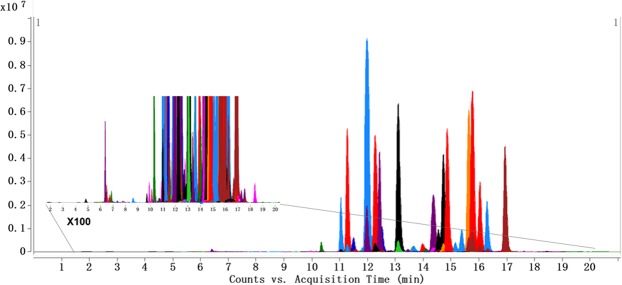
MRM chromatograms of 87 sphingolipids in human serum.

The sphingolipids in human serum were comprehensively profiled using an established sphingolipidomic approach^23^. A total of 87 sphingolipids were identified and quantified in the serum samples, including 9 sphingosines, 3 sphinganines (Sas), S1P, 19 Cers, 1 ceramide-1-phosphate (Cer1P), 44 sphingomyelins (SMs), 4 hexosylceramides (HexCers), and 6 lactosylceramides (LacCers). The MS and MS/MS data of identified sphingolipids and the multiple reaction monitoring (MRM) transitions used to monitor each sphingolipid are listed in Table 2. The MRM chromatograms of all sphingolipids in human serum are shown in Fig. 1.

**Table 2:** Identification and quantification of 87 sphingolipids in human serum using UHPLC-Q-TOF and UHPLC-QQQ MS.

Q-TOF MS								QQQ MS		
Subclass	Name	RT (min)	Molecular Formula	Measured *m/z*	Calculated *m/z*	Error (ppm)	MS/MS Fragments (*m/z*)	MRM transition	Fragmentor (V)	CE (V)
So	So (d17:1) [I.S.-1]	6.34	C_17_H_35_NO_2_	286.2748	286.2741	2.45	268.2649	286.4 → 268.2	80	5
	So (m18:2)	8.59	C_18_H_35_NO	282.2791	282.2791	0.00	264.2691	282.3 → 264.3	80	5
	So (m18:3)	7.79	C_18_H_33_NO	280.2639	280.2635	1.43	262.2542	280.3 → 262.3	80	5
	So (d16:1)	4.85	C_16_H_33_NO_2_	272.2583	272.2584	−0.37	254.2833	272.3 → 254.3	80	5
	So (d18:1)	6.71	C_18_H_37_NO_2_	300.2899	300.2897	0.67	282.2797	300.3 → 282.3	80	5
	So (d18:2)	6.78	C_18_H_35_NO_2_	298.2743	298.2741	0.67	280.2630	298.3 → 280.3	80	5
	So (d18:3)	6.34	C_18_H_33_NO_2_	296.2586	296.2584	0.68	278.2473	296.3 → 278.2	80	5
	So (t18:1)	7.32	C_18_H_37_NO_3_	316.2841	316.2846	−1.58	298.2733	316.3 → 298.3	80	5
	So (d22:3)	7.91	C_22_H_41_NO_2_	352.3205	352.3210	−1.42	334.3101	352.3 → 334.3	80	5
	So (d22:3) isomer	7.62	C_22_H_41_NO_2_	352.3219	352.3210	2.55	334.3100	352.3 → 334.3	80	5
Sa	Sa (d17:0) [I.S.-2]	6.57	C_17_H_37_NO_2_	288.2901	288.2897	1.39	270.2795, 60.0453	288.4 → 270.2	110	20
	Sa (d16:0)	5.03	C_16_H_35_NO_2_	274.2743	274.2741	0.73	256.2653	274.3 → 256.3	110	20
	Sa (d18:0)	6.34	C_18_H_39_NO_2_	302.3056	302.3054	0.66	284.2950	302.3 → 284.3	110	20
	Sa (d20:0)	6.79	C_20_H_43_NO_2_	330.3376	330.3367	2.72	312.3268	330.3 → 312.3	110	20
S1P	S1P (d17:1) [I.S.-3]	6.49	C_17_H_36_NO_5_P	366.2406	366.2404	0.55	250.2508	366.3 → 250.3	105	10
	S1P (d18:1)	6.90	C_18_H_38_NO_5_P	380.2578	380.2560	4.73	264.2690	380.3 → 264.3	105	10
	Sa1P (d17:0) [I.S.-4]	6.71	C_17_H_38_NO_5_P	368.2571	368.2560	2.99	270.2788	368.4 → 270.3	120	5
Cer	Cer (d18:1/12:0) [I.S.-5]	10.92	C_30_H_59_NO_3_	482.4582	482.4568	2.90	464.4463, 282.2790, 264.2691	482.6 → 264.3	130	25
	Cer (d18:0/16:0)	13.75	C_34_H_69_NO_3_	540.5349	540.5350	−0.19	266.2837	540.5 → 266.3	130	25
	Cer (d18:0/18:0)	14.30	C_36_H_73_NO_3_	568.5674	568.5663	1.93	266.2831	568.6 → 266.3	130	25
	Cer (d18:0/22:0)	17.00	C_40_H_81_NO_3_	624.6278	624.6289	−1.76	606.6, 284.2938, 266.2833	624.6 → 266.3	130	25
	Cer (d18:0/23:0)	18.20	C_41_H_83_NO_3_	638.6446	638.6446	0.00	620.6307, 284.2939, 266.2846	638.6 → 266.3	130	25
	Cer (d18:0/24:0)	19.14	C_42_H_85_NO_3_	652.6597	652.6602	−0.77	634.6402, 284.2925, 266.2838	652.7 → 266.3	130	25
	Cer (d18:1/16:0)	13.06	C_34_H_67_NO_3_	538.5194	538.5194	0.00	520.5010, 282.2796, 264.2691	538.5 → 264.3	130	25
	Cer (d18:1/18:0)	14.20	C_36_H_71_NO_3_	566.5503	566.5507	−0.71	548.5406, 264.2690	566.6 → 264.3	130	25
	Cer (d18:1/18:1)	13.06	C_36_H_69_NO_3_	564.5340	564.5350	−1.77	264.2685	564.5 → 264.3	130	25
	Cer (d18:1/20:1)	14.34	C_38_H_73_NO_3_	592.5670	592.5663	1.18	264.2688	592.6 → 264.3	130	25
	Cer (d18:1/22:0)	17.01	C_40_H_79_NO_3_	622.6133	622.6133	0.00	604.6019, 264.2691	622.6 → 264.3	130	25
	Cer (d18:1/23:0)	17.72	C_41_H_81_NO_3_	636.6285	636.6289	−0.63	618.6185, 264.2689	636.6 → 264.3	130	25
	Cer (d18:1/24:0)	18.51	C_42_H_83_NO_3_	650.6448	650.6446	0.31	632.6343, 282.2783, 264.2691	650.6 → 264.3	130	25
	Cer (d18:1/24:1)	17.02	C_42_H_81_NO_3_	648.6280	648.6289	−1.39	630.6188, 264.2688	648.6 → 264.3	130	25
	Cer (d18:1/25:0)	19.05	C_43_H_85_NO_3_	664.6596	664.6602	−0.90	646.6434, 282.2795, 264.2695	664.7 → 264.3	130	25
	Cer (d18:1/25:0) isomer	19.41	C_43_H_85_NO_3_	664.6598	664.6602	−0.60	646.6434, 282.2795, 264.2695	664.7 → 264.3	130	25
	Cer (d18:1/26:0)	20.45	C_44_H_87_NO_3_	678.6741	678.6759	−2.65	660.6650, 264.2695	678.7 → 264.3	130	25
	Cer (d18:2/16:0)	12.41	C_34_H_65_NO_3_	536.5044	536.5037	1.30	262.2549	536.5 → 262.3	130	25
	Cer (d18:2/22:0)	16.08	C_40_H_77_NO_3_	620.5980	620.5976	0.64	602.5875, 262.2546	620.6 → 262.2	130	25
	Cer (d18:2/23:0)	16.77	C_41_H_79_NO_3_	634.6115	634.6133	−2.84	262.2542	634.6 → 262.3	130	25
	Cer1P (d18:1/12:0) [I.S.-6]	9.95	C_30_H_60_NO_6_P	562.4242	562.4231	1.96	264.2615	562.5 → 264.3	135	25
	Cer1P (d18:1/18:0)	12.78	C_36_H_72_NO_6_P	646.5167	646.5170	−0.46	264.2611	646.5 → 264.3	135	25
SM	SM (d18:1/12:0) [I.S.-7]	10.30	C_35_H_71_N_2_O_6_P	647.5124	647.5123	0.15	264.2704, 184.0741	647.5 → 184.1	170	20
	SM (d18:0/18:0)	13.65	C_41_H_85_N_2_O_6_P	733.6217	733.6218	−0.14	184.0741	733.6 → 184.1	170	20
	SM (d18:1/14:0)	11.08	C_37_H_75_N_2_O_6_P	675.5420	675.5436	−2.37	657.5282, 264.2670, 184.0758	675.5 → 184.1	170	20
	SM (d18:1/16:0)	12.03	C_39_H_79_N_2_O_6_P	703.5735	703.5749	−1.99	685.5649, 264.2703, 184.0734	703.6 → 184.1	170	20
	SM (d18:1/17:1)	11.75	C_40_H_79_N_2_O_6_P	715.5747	715.5749	−0.28	264.2670, 184.0759	715.6 → 184.1	170	20
	SM (d18:1/18:0)	13.10	C_41_H_83_N_2_O_6_P	731.6056	731.6062	−0.82	713.5884, 264.2702, 184.0762	731.6 → 184.1	170	20
	SM (d18:1/18:2)	11.60	C_41_H_79_N_2_O_6_P	727.5737	727.5749	−1.65	264.2671, 184.0749	727.6 → 184.1	170	20
	SM (d18:1/20:0)	14.37	C_43_H_87_N_2_O_6_P	759.6356	759.6375	−2.50	741.6249, 264.2674, 184.0757	759.6 → 184.1	170	20
	SM (d18:1/22:0)	15.60	C_45_H_91_N_2_O_6_P	787.6680	787.6688	−1.02	769.6525, 264.2690, 184.0761	787.7 → 184.1	170	20
	SM (d18:1/23:0)	16.36	C_46_H_93_N_2_O_6_P	801.6833	801.6844	−1.37	264.2675, 184.0744	801.7 → 184.1	170	20
	SM (d18:1/23:1)	15.18	C_46_H_91_N_2_O_6_P	799.6673	799.6688	−1.88	264.2680, 184.0764	799.7 → 184.1	170	20
	SM (d18:1/24:0)	16.90	C_47_H_95_N_2_O_6_P	815.6989	815.7001	−1.47	264.2612, 184.0729	815.7 → 184.1	170	20
	SM (d18:1/24:1)	16.10	C_47_H_93_N_2_O_6_P	813.6825	813.6844	−2.34	795.6724, 264.2606, 184.0759	813.7 → 184.1	170	20
	SM (d18:1/26:1)	17.02	C_49_H_97_N_2_O_6_P	841.7166	841.7157	1.07	264.2602, 184.0761	841.7 → 184.1	170	20
	SM (d18:2/16:0)	11.31	C_39_H_77_N_2_O_6_P	701.5585	701.5592	−1.00	683.5426, 262.2520, 184.0763	701.6 → 184.1	170	20
	SM (d18:2/16:1)	10.79	C_39_H_75_N_2_O_6_P	699.5423	699.5436	−1.86	262.2523, 184.0765	699.5 → 184.1	170	20
	SM (d18:2/18:0)	12.31	C_41_H_81_N_2_O_6_P	729.5893	729.5905	−1.64	711.5798, 262.2545, 184.0761	729.6 → 184.1	170	20
	SM (d16:1/24:1)	14.50	C_45_H_89_N_2_O_6_P	785.6548	785.6531	2.16	236.2379, 184.0744	785.7 → 184.1	170	20
	SM (d18:2/22:0)	14.70	C_45_H_89_N_2_O_6_P	785.6545	785.6531	1.78	262.2533, 184.0742	785.7 → 184.1	170	20
	SM (d18:2/22:1)	13.60	C_45_H_87_N_2_O_6_P	783.6383	783.6375	1.02	262.2536, 184.0743	783.6 → 184.1	170	20
	SM (d18:2/23:0)	15.41	C_46_H_91_N_2_O_6_P	799.6710	799.6688	2.75	262.2535, 184.0745	799.7 → 184.1	170	20
	SM (d18:2/24:0)	15.77	C_47_H_93_N_2_O_6_P	813.6855	813.6844	1.35	262.2527, 184.0743	813.7 → 184.1	170	20
	SM (d18:2/24:1)	14.80	C_47_H_91_N_2_O_6_P	811.6701	811.6688	1.60	262.2535, 184.0743	811.7 → 184.1	170	20
	SM (d18:2/25:0)	16.21	C_48_H_95_N_2_O_6_P	827.6995	827.7001	−0.72	262.2548, 184.0741	827.7 → 184.1	170	20
	SM (d18:2/25:0) isomer-1	16.42	C_48_H_95_N_2_O_6_P	827.6989	827.7001	−1.45	262.2548, 184.0741	827.7 → 184.1	170	20
	SM (d18:2/25:0) isomer-2	16.72	C_48_H_95_N_2_O_6_P	827.7022	827.7001	2.54	262.2548, 184.0741	827.7 → 184.1	170	20
	SM (d32:0)	11.43	C_37_H_77_N_2_O_6_P	677.5580	677.5592	−1.77	184.0740	677.6 → 184.1	170	20
	SM (d33:0)	11.90	C_38_H_79_N_2_O_6_P	691.5761	691.5749	1.74	184.0739	691.6 → 184.1	170	20
	SM (d33:1)	11.51	C_38_H_77_N_2_O_6_P	689.5606	689.5592	2.03	184.0744	689.6 → 184.1	170	20
	SM (d34:0)	12.43	C_39_H_81_N_2_O_6_P	705.5924	705.5905	2.69	184.0745	705.6 → 184.1	170	20
	SM (d35:0)	13.20	C_40_H_83_N_2_O_6_P	719.6050	719.6062	−1.67	184.0744	719.6 → 184.1	170	20
	SM (d35:1)	12.54	C_40_H_81_N_2_O_6_P	717.5914	717.5905	1.25	184.0741	717.6 → 184.1	170	20
	SM (d35:1) isomer	12.37	C_40_H_81_N_2_O_6_P	717.5918	717.5905	1.81	184.0754	717.6 → 184.1	170	20
	SM (d37:1)	13.76	C_42_H_85_N_2_O_6_P	745.6223	745.6218	0.67	184.0740	745.6 → 184.1	170	20
	SM (d37:2)	12.85	C_42_H_83_N_2_O_6_P	743.6081	743.6062	2.56	184.0733	743.6 → 184.1	170	20
	SM (d38:0)	14.90	C_43_H_89_N_2_O_6_P	761.6530	761.6531	−0.13	184.0746	761.7 → 184.1	170	20
	SM (d38:2)	13.46	C_43_H_85_N_2_O_6_P	757.6231	757.6218	1.72	184.0745	757.6 → 184.1	170	20
	SM (d39:2)	14.09	C_44_H_87_N_2_O_6_P	771.6387	771.6375	1.56	184.0734	771.6 → 184.1	170	20
	SM (d40:0)	16.10	C_45_H_93_N_2_O_6_P	789.6847	789.6844	0.38	184.0742	789.7 → 184.1	170	20
	SM (d41:0)	16.85	C_46_H_95_N_2_O_6_P	803.7001	803.7001	0.00	184.0739	803.7 → 184.1	170	20
	SM (d41:3)	14.31	C_46_H_89_N_2_O_6_P	797.6529	797.6531	−0.25	184.0737	797.7 → 184.1	170	20
	SM (d42:4)	14.00	C_47_H_89_N_2_O_6_P	809.6553	809.6531	2.72	184.0744	809.7 → 184.1	170	20
	SM (d43:1)	17.62	C_48_H_97_N_2_O_6_P	829.7169	829.7157	1.45	184.0747	829.7 → 184.1	170	20
	SM (d43:1) isomer	17.35	C_48_H_97_N_2_O_6_P	829.7165	829.7157	0.96	184.0728	829.7 → 184.1	170	20
	SM (t44:4)	14.82	C_49_H_93_N_2_O_7_P	853.6787	853.6793	−0.70	184.0730	853.7 → 184.1	170	20
HexCer	GalCer (d18:1/12:0) [I.S.-8]	10.34	C_36_H_69_NO_8_	644.5107	644.5096	1.71	264.2684	644.5 → 264.3	130	30
	HexCer (d18:1/20:0)	14.30	C_44_H_85_NO_8_	756.6327	756.6348	−2.78	264.2685	756.6 → 264.3	130	30
	HexCer (d18:1/22:0)	15.60	C_46_H_89_NO_8_	784.6659	784.6661	−0.25	264.2689	784.7 → 264.3	130	30
	HexCer (d18:1/23:0)	16.30	C_47_H_91_NO_8_	798.6805	798.6817	−1.50	264.2676	798.7 → 264.3	130	30
	HexCer (d18:1/24:0)	16.90	C_48_H_93_NO_8_	812.6973	812.6974	−0.12	264.2685	812.7 → 264.3	130	30
	LacCer (d18:1/12:0) [I.S.-9]	10.13	C_42_H_79_NO_13_	806.5640	806.5624	1.98	788.5498, 482.4589, 264.2683	806.7 → 264.3	145	40
	LacCer (d18:1/16:0)	10.96	C_46_H_87_NO_13_	862.6259	862.6250	1.04	844.6139, 538.5174, 264.2687	862.6 → 264.3	145	40
	LacCer (d18:1/18:0)	11.33	C_48_H_91_NO_13_	890.6549	890.6563	−1.57	264.2696	890.7 → 264.3	145	40
	LacCer (d18:1/20:0)	16.95	C_50_H_95_NO_13_	918.6860	918.6876	−1.74	264.2688	918.7 → 264.3	145	40
	LacCer (d18:1/22:0)	15.00	C_52_H_99_NO_13_	946.7192	946.7189	0.32	264.2679	946.7 → 264.3	145	40
	LacCer (d18:1/24:0)	16.30	C_54_H_103_NO_13_	974.7512	974.7502	1.03	264.2672	974.8 → 264.3	145	40
	LacCer (d18:1/24:1)	15.10	C_54_H_101_NO_13_	972.7323	972.7346	−2.36	264.2650	972.7 → 264.3	145	40

Notes: Abbreviations: MRM, multiple reaction monitoring; RT, retention time; CE, collision energy; So, sphingosine; Sa, sphinganine; S1P, sphingosine-1-phosphate; Cer, ceramide; Cer1P, ceramide-1-phosphate; SM, sphingomyelin; GalCer, galactosylceramide; HexCer, hexosylceramide; LacCer, lactosylceramide. I.S. -1–9 represent the nine internal standards added for MS analysis.

### Differences in serum sphingolipidomes between PCOS patients and healthy women

**Figure 2:**
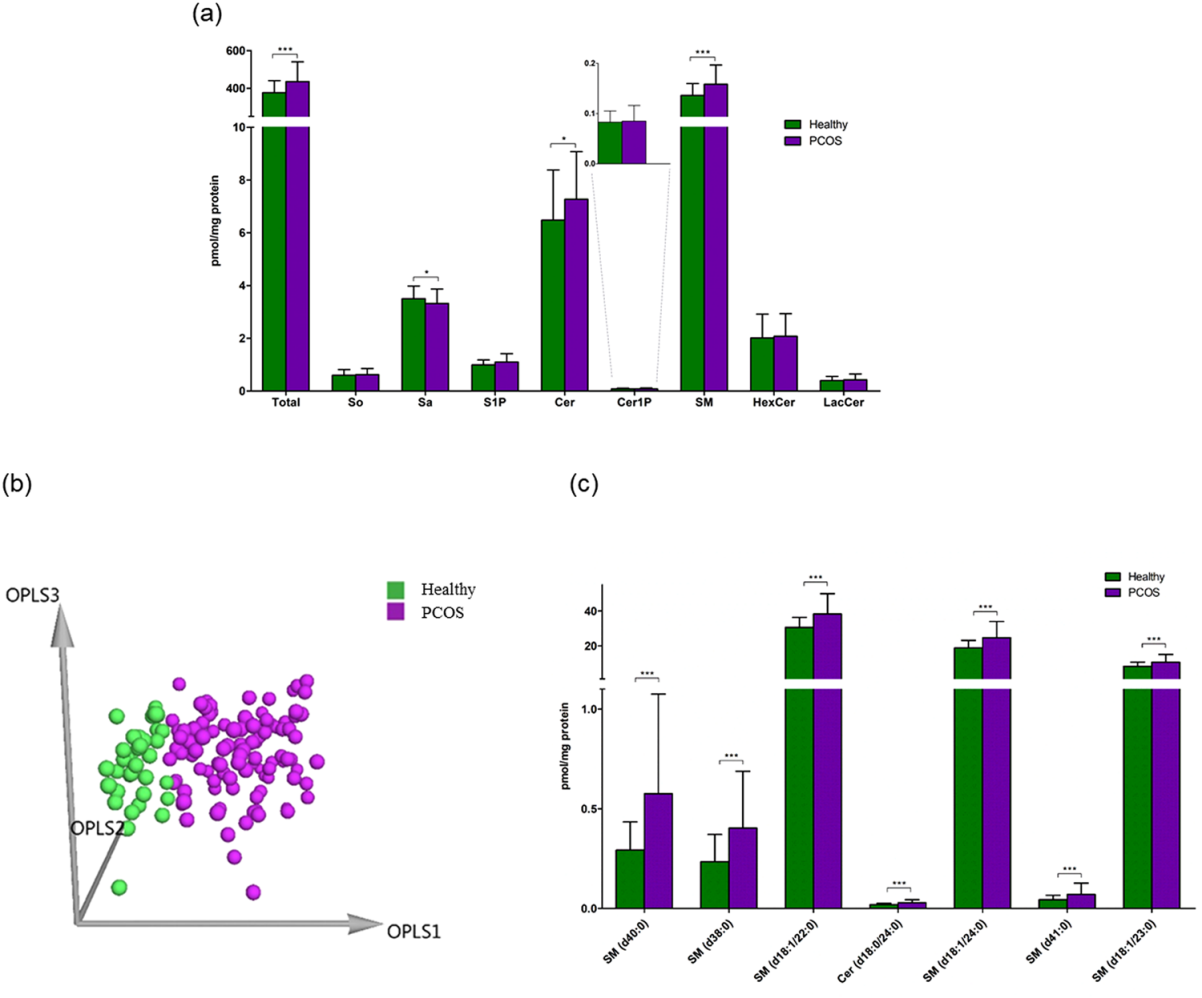
(**a**) The levels of total sphingolipids and of each subclass of sphingolipid in healthy women (*n* = 37) and PCOS patients (*n* = 107). Each column represents the mean ± SD (*p < 0.05, ***p < 0.001). So, sphingosine; Sa, sphinganine; S1P, sphingosine-1-phosphate; Cer, ceramide; Cer1P, ceramide-1-phosphate; SM, sphingomyelin; HexCer, hexosylceramide; LacCer, lactosylceramide. (**b**) The 3D OPLS-DA score plot for the healthy group (*n* = 37) and the PCOS group (*n* = 107) (R^2^X = 0.523, R^2^Y = 0.697, Q^2^ = 0.533). (**c**) The potential biomarkers (SM (d40:0), SM (d38:0), SM (d18:1/22:0), Cer (d18:0/24:0), SM (d18:1/24:0), SM (d41:0), and SM (d18:1/23:0)) for the classification between the healthy group and the PCOS group. Each column represents the mean ± SD (***p < 0.001).

As shown in Fig. 2a, the total sphingolipid content in PCOS patients showed a significant increase (15.9% increase) when compared with healthy women. Among the sphingolipids, three subclasses demonstrated remarkable elevations in PCOS patients, including S1P (10.8% increase), Cer (12.2% increase), and SM (16.3% increase). Sa was the only subclass showing a decrease in PCOS patients. Multivariate analysis was subsequently carried out to discriminate between PCOS patients and healthy women. As shown in Fig. 2b, the three-dimensional (3D) orthogonal partial least squares discriminant analysis (OPLS-DA) score plot demonstrated a clear separation between the PCOS group and the healthy group (R^2^X = 0.523, R^2^Y = 0.697, Q^2^ = 0.533). Sphingolipids with a variable importance plot (VIP) value > 1.5 were regarded as the important variables contributing to the 3D OPLS-DA model. As a result, 7 sphingolipids, including 6 SMs (SM (d40:0), SM (d38:0), SM (d18:1/22:0), SM (d18:1/24:0), SM (d41:0), and SM (d18:1/23:0)) and 1 Cer (Cer (d18:0/24:0)) were selected as potential biomarkers (Fig. 2c).

### Differences in serum sphingolipidomes between different subgroups of PCOS and healthy women

**Figure 3:**
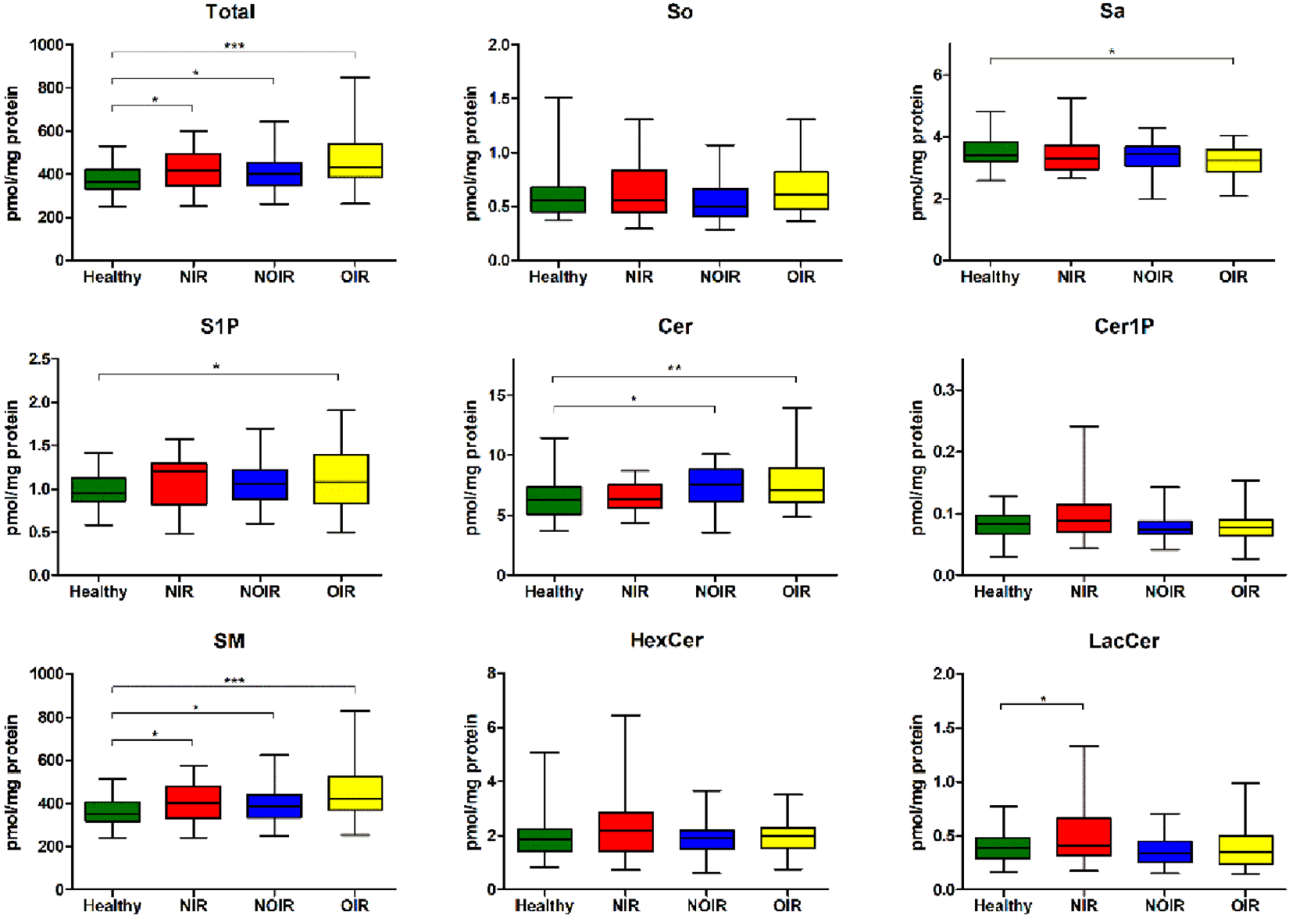
Box and whiskers plot showing the levels of total sphingolipids and of each subclass of sphingolipid in healthy women (*n* = 37), NIR PCOS patients (*n* = 32), NOIR PCOS patients (*n* = 34), and OIR PCOS patients (*n* = 41). *p < 0.05, **p < 0.01, ***p < 0.001. So, sphingosine; Sa, sphinganine; S1P, sphingosine-1-phosphate; Cer, ceramide; Cer1P, ceramide-1-phosphate; SM, sphingomyelin; HexCer, hexosylceramide; LacCer, lactosylceramide.

The PCOS group was classified into three subgroups (OIR, NOIR, NIR) as described above for further comparison with the healthy group. As shown in Fig. 3, the levels of total sphingolipids and of each sphingolipid subclass in all three subgroups of PCOS followed similar trends as the overall PCOS group. Notably, OIR showed the most obvious elevation (23.83%) in the total sphingolipid level when compared to the healthy group. All three subgroups of PCOS demonstrated significant increases in the level of SM (OIR > NOIR > NIR), while only NOIR and OIR showed significant increases in the level of Cer (OIR > NOIR), and only OIR showed significant increases in the level of S1P. The level of Sa was decreased in all three subgroups of PCOS, in which only OIR demonstrated a significant change. Unexpectedly, the level of LacCer was only elevated in NIR (28.72%), and there was almost no change in NOIR and OIR.

### Differences in serum sphingolipidomes among different subgroups of PCOS patients

**Figure 4:**
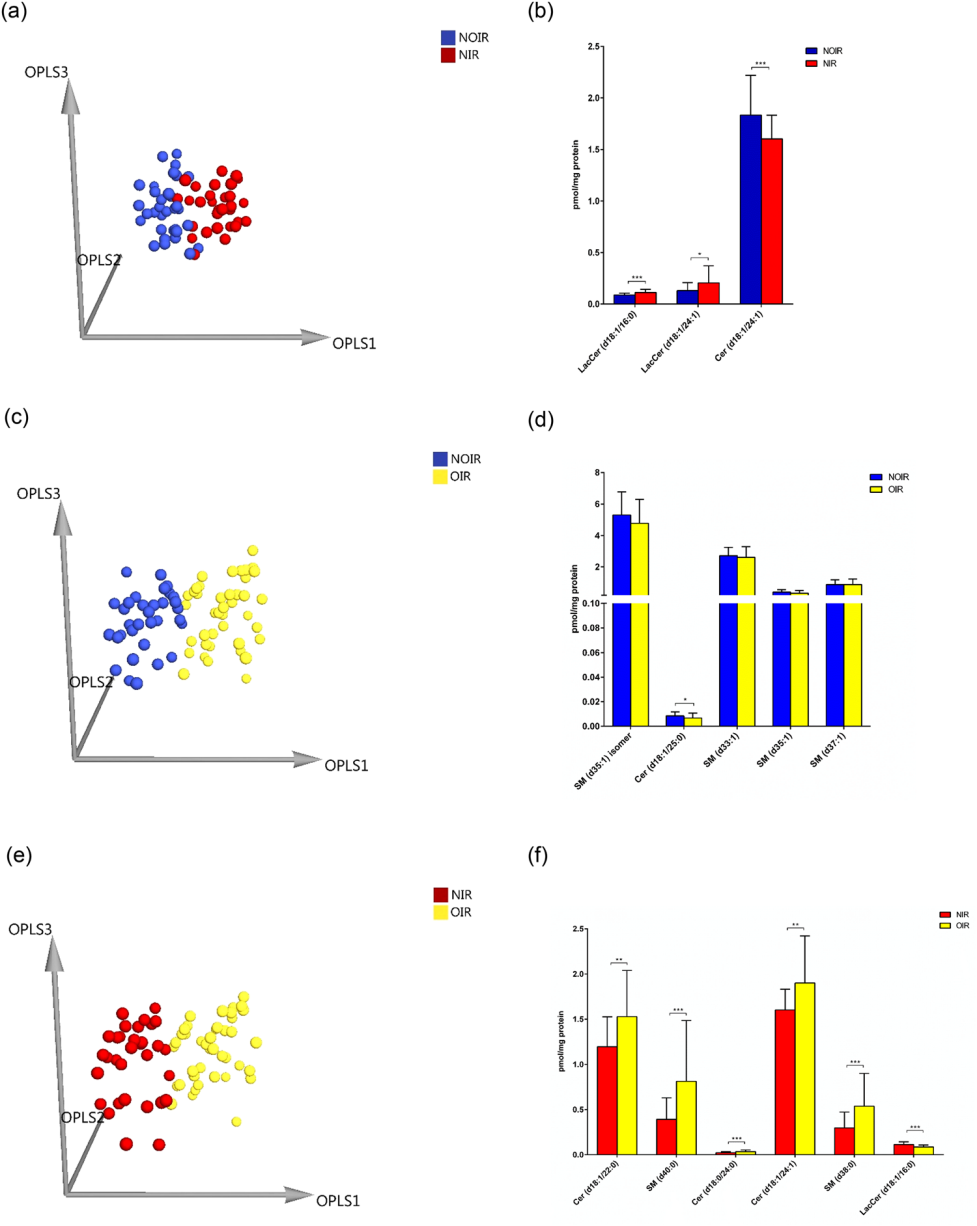
(**a**) The 3D OPLS-DA score plot for NOIR patients (*n* = 34) and NIR patients (*n* = 32) (R^2^X = 0.499, R^2^Y = 0.691, Q^2^ = 0.301). (**b**) The potential markers (LacCer (d18:1/16:0), LacCer (d18:1/24:1), and Cer (d18:1/24:1)) for the classification between NOIR patients and NIR patients. Each column represents the mean ± SD. *p < 0.05, ***p < 0.001. (**c**) The 3D OPLS-DA score plot for NOIR patients (*n* = 34) and OIR patients (*n* = 41) (R^2^X = 0.481, R^2^Y = 0.415, Q^2^ = 0.113). (**d**) The potential marker (Cer (d18:1/25:0)) for the classification between NOIR patients and OIR patients. The columns represent the mean ± SD. *p < 0.05. (**e**) The 3D OPLS-DA score plot for NIR patients (*n* = 32) and OIR patients (*n* = 41) (R^2^X = 0.516, R^2^Y = 0.534, Q^2^ = 0.416). (**f**) The potential markers (Cer (d18:1/22:0), SM (d40:0), Cer (d18:0/24:0), Cer (d18:1/24:1), SM (d38:0), and LacCer (d18:1/16:0)) for the classification between NIR patients and OIR patients. Each column represents the mean ± SD. **p < 0.01, ***p < 0.001.

Multivariate statistical analysis was carried out to discriminate between NIR, NOIR, and OIR. Figure 4a shows the 3D OPLS-DA score plot of NOIR and NIR (R^2^X = 0.499, R^2^Y = 0.691, Q^2^ = 0.301), and 3 sphingolipids, including 2 LacCers (LacCer (d18:1/16:0) and LacCer (d18:1/24:1)) and 1 Cer (Cer (d18:1/24:1)), were identified as potential markers (Fig. 4b). Figure 4c shows the 3D OPLS-DA score plot of NOIR and OIR (R^2^X = 0.481, R^2^Y = 0.415, Q^2^ = 0.113), and 1 Cer (Cer (d18:1/25:0)) was identified as a potential marker (Fig. 4d). Figure 4e shows the 3D OPLS-DA score plot of NIR and OIR (R^2^X = 0.516, R^2^Y = 0.534, Q^2^ = 0.416), and 6 sphingolipids, including 3 Cers (Cer (d18:1/22:0), Cer (d18:0/24:0), and Cer (d18:1/24:1)), 2 SMs (SM (d40:0) and SM (d38:0)), and 1 LacCer (LacCer (d18:1/16:0)) were identified as potential markers (Fig. 4f).

## Discussion

In this study, the serum sphingolipids in healthy controls and PCOS patients were comprehensively investigated using the improved LC-MS–based sphingolipidomic approach^23^. As a result, 87 sphingolipids were identified and quantified in each sample. Notable elevations in the levels of S1P, Cer, and SM were observed in PCOS patients, and these made the largest contribution to the significant increase in the total sphingolipid content in PCOS patients. Our results are in accordance with a previous study that suggested that PCOS is associated with increased TG and SM and decreased phosphatidylethanolamines and lysophosphatidylcholines in patient plasma^35^. The multivariate analysis between PCOS patients and healthy women also suggested that SM was the most dominant subclass of sphingolipid involved in the pathogenesis of PCOS because 6 out of 7 potential markers were SMs. Notably, all potential markers, including the one Cer marker, have saturated acyl chains. A previous report showed that the increase in SM species with long saturated acyl chains (18:0, 20:0, 22:0, and 24:0) was closely correlated with the development of metabolic syndrome associated with lipid metabolism, obesity, and IR^36^. Therefore, we suggest that SM species with long saturated acyl chains might serve as novel biomarkers of PCOS, and further studies should be carried out to confirm this.

In addition to PCOS, other metabolic diseases such as obesity and IR have also been shown to be associated with lipid alterations^37^. Therefore, we further divided the PCOS patients into three subgroups (NIR, NOIR, OIR) according to their HOMA-IR and BMI values. NIR represented the group without IR or obesity, NOIR represented the group with IR only, and OIR represented the group with both IR and obesity. The levels of total sphingolipids and of each subclass in all three subgroups of PCOS followed similar trends as for all PCOS patients combined, while the subgroups of PCOS with IR or obesity exhibited greater changes than their corresponding subgroups without IR or obesity. As mentioned above, an increase in SM species with long saturated acyl chains was closely associated with lipid metabolism, obesity, and IR. Therefore, the presence of IR or both IR and obesity contributed to the increase in the level of SM in the order of OIR > NOIR > NIR. In addition, plasma Cer was reported to be elevated in obese subjects with type 2 diabetes mellitus, and increased levels of plasma Cer might also be a marker of IR^38^. However, in our study the occurrence of PCOS seems to have no direct relationship with Cer, and only NOIR and OIR showed significant increases in the level of Cer (OIR > NOIR). Furthermore, S1P was also reported to be elevated in the plasma of obese subjects and to correlate with IR^39^, and this is the likely explanation for why only OIR demonstrated a significant increase in the level of S1P in our study.

No previous report has discussed the relation between Sa and PCOS. However, because Sa is upstream of Cer in the metabolism pathway of sphingolipids, we speculate that the decrease we observed in Sa might be the result of the upregulation of Cer synthases leading to the accumulation of Cer and further downstream products. However, further studies are needed to confirm this hypothesis.

It is interesting that the level of LacCer was only elevated in NIR, while there was almost no change in NOIR and OIR. This implies that the level of LacCer was significantly increased in PCOS patients who did not suffer from IR and obesity. However, previous studies indicated that there is a negative correlation between gangliosides (complex glycosphingolipids) and insulin responsiveness^40^, and it was observed that inhibition of glucosylceramide synthase activity reversed IR in several rodent models of obesity^41^. Therefore, in theory the level of LacCer would be expected to be increased in PCOS patients with IR. However, up to now there have been no studies to investigate the direct interaction of LacCer on insulin responsiveness and the relationship between glucosylceramide synthase activity and IR in humans. Thus the results of the subgroup analysis further suggest that PCOS patients have unique sphingolipid biomarkers that are not caused only by obesity and IR.

In the multivariate statistical analysis of sphingolipid alterations among the three subgroups of PCOS, it was found that the discrimination between the three subgroups of PCOS was unsatisfactory, especially between NOIR and OIR. Only a single sphingolipid showed a significant difference between NOIR and OIR, and this might be explained by the close relationship between IR and obesity, which often cause similar alterations in sphingolipid metabolism. The differentiation between NIR and OIR was comparatively better. These results suggested that alterations in sphingolipid metabolism might play a role in the pathogenesis of PCOS and that sphingolipids might be useful as diagnostic biomarkers for different types of PCOS.

This study represents the first report of a comprehensive sphingolipidomic profiling of PCOS and different subgroups of PCOS with or without IR and/or obesity. The results shed light on the diagnosis and pathogenesis of PCOS. A limitation of the study is that we only detected some of the sphingolipid characteristics for different types of PCOS patients, and the mechanism of alterations of sphingolipid metabolism in PCOS needs further investigation in both human subjects and animal models.

## Materials and Methods

### Study Participants

PCOS patients aged 18 to 40 years were recruited between January 2014 and May 2015 from the Department of Traditional Chinese Medicine of the First Affiliated Hospital of Guangzhou Medical University. The first woman was recruited on January 2, 2014, and the last one was on May 28, 2015. Age-matched healthy female subjects were selected from community volunteers. PCOS was diagnosed as having two of the following three Rotterdam criteria^42^: (1) oligomenorrhea/amenorrhea; (2) more than 12 follicles ≤9 mm in diameter or ovarian volume >10 ml on pelvic scanning; and (3) clinical or biochemical hyperandrogenism. Oligomenorrhea is defined as an intermenstrual interval >35 days or <8 menstrual bleedings in the past year. Amenorrhea is defined as an intermenstrual interval >90 days. Biochemical hyperandrogenism is defined as a TT level ≥ 0.6 ng/ml^43^, and clinical hyperandrogenism is defined as a Ferriman–Gallwey score ≥ 5^44^. Women were excluded if they had other endocrine disorders such as hyperprolactinemia, type I or type II diabetes mellitus, non-classic congenital adrenal hyperplasia, suspected Cushing’s syndrome, androgen-secreting tumors, thyroid diseases, or drug-induced androgen excess. Women who had received any hormonal treatments, Chinese herbal prescriptions, or acupuncture treatments in the past 3 months were also excluded from the study. All controls had regular menstrual cycles and normal hormone levels.

Eligible PCOS patients were further categorized as obese if they had a BMI ≥ 25 (kg/m^2^) according to the World Health organization (WHO) criteria for Asians^45^ and were categorized as insulin resistant if they had a HOMA-IR (calculated as [fasting glucose] × [fasting insulin]/22.5)) ≥ 2.14^46^.

This study was approved by the ethics committee of the First Affiliated Hospital of Guangzhou Medical University (No. 2013–39 and 2016–64). Informed written consent was obtained from all subjects before inclusion.

### Body conditions and detection of substances related to endocrine, glucose, and lipid metabolism

The height, weight, waist circumference, hip circumference, and hirsutism scores of all women were recorded, and blood samples were collected in the first 2–5 days of their menstrual cycle at 08.00–10.00 in the morning after fasting for 12 hours. The serum samples were batched and analyzed in the laboratory in the First Affiliated Hospital of Guangzhou Medical University, and serum FSH, LH, estradiol (E2), TT, prolactin, TG, CHOL, LDL, HDL, fasting plasma glucose, and fasting insulin were measured. The intra-assay and inter-assay coefficients of variation were less than 5%. HOMA-IR was calculated to assess changes in insulin sensitivity^40^. FSH, LH, and TT levels were measured with a Beckman-Coulter Unicel DXi800 automatic chemiluminescence analyzer (Beckman Coulter, Brea, USA), and TG, CHOL, LDL, and HDL levels were measured on a Beckman-Coulter AU5800 automatic biochemical analyzer. Fasting insulin was analyzed using the Modular E170® automatic electrochemiluminescent analyzer (Roche Diagnostics, Mannheim, Germany). Fasting plasma glucose was measured on a Beckman Coulter LX20 automatic biochemical analyzer. All assays were performed based on the instructions of manufacturers and with reagents and materials provide by the manufacturers.

### Sample collection and processing

All of the blood samples were collected by staff in the First Affiliated Hospital of Guangzhou Medical University. The blood samples were 5 ml and were centrifuged for 15 minutes at 3,000 rpm within 30 minutes after the blood samples were drawn. The separated serum was stored at −80 °C and was delivered to the State Key Laboratory of Quality Research in Chinese Medicine, Macau University of Science and Technology, within 3 months of being collected.

### Sample preparation and sphingolipidomic assays

#### Extraction of sphingolipids

Serum sphingolipids were extracted using an established method^23^. Briefly, 20 μl of serum was transferred into a borosilicate glass tube, and 10 μl of internal standards (2.5 μM each) and 0.75 ml of extraction solvent [methanol (MeOH): chloroform, 2:1, v/v] were added. The contents were dispersed in an ultrasonicator at room temperature for 30 seconds, followed by incubation at 48 °C for 12 hours. After cooling, 75 μl of potassium hydroxide in MeOH (1 M) was added and incubated with shaking at 37 °C for 2 hours, after which 3 μl acetic acid was added to neutralize the extract. After centrifugation, the supernatant was stored in a 4 ml bottle, and the residue was re-extracted twice. Finally, the extract was dried by nitrogen and re-dissolved in 150 μl of MeOH for liquid chromatography-mass spectrometry (LC-MS) detection. The quality control (QC) sample was pooled with equal quantities of samples from the different groups for the sphingolipid analysis.

#### Detection of sphingolipids by LC-MS

Serum sphingolipids were detected with the improved LC-MS–based sphingolipidomic approach^23^. The chromatographic separation of sphingolipids was performed on an Agilent 1290 Infinity UHPLC system (Santa Clara, CA, USA) with an Agilent Eclipse Plus C_18_ column (100 mm × 2.1 mm, 1.8 μm) at 40 °C. The mobile phase consisted of (A) MeOH/H_2_O/formic acid (HCOOH) (60:40:0.2, v/v/v) and (B) MeOH/isopropyl alcohol/HCOOH (60:40:0.2, v/v/v), both containing 10 mM NH_4_OAc. Qualitative analysis of sphingolipids was performed on an Agilent ultra high definition (UHD) 6550 quadrupole time-of-flight (Q-TOF) MS (Santa Clara, CA, USA). The MS source parameters were as follows: drying gas (nitrogen) temperature 200 °C, drying gas flow 11 L/min, sheath gas (nitrogen) temperature 300 °C, sheath gas flow 12 L/min, capillary voltage 4000 V, nozzle voltage 200 V, nebulizer pressure 40 psi, skimmer voltage 65 V, octopole RF peak 500 V, and fragmentor voltage 175 V. The targeted MS/MS collision energy was set at 20–60 eV. MS spectra and MS-MS spectra were acquired in positive mode with the mass range of *m/z* 110–1700 and *m/z* 40–1700, respectively. A reference solution was nebulized for continuous calibration using the reference mass of *m/z* 922.0098. Quantitative analysis of sphingolipids was carried out on an Agilent 6460 triple quadrupole (QQQ) MS. The MS source parameters were as follows: drying gas (nitrogen) temperature 325 °C, drying gas flow 11 L/min, capillary voltage 4000 V, and nebulizer pressure 30 psi. The optimized parameters for each individual sphingolipid, such as characteristic transition (precursor ion → product ion), fragmentor voltage, and collision energy are shown in Table 2.

#### Data analysis

An in-house sphingolipid database has been established in our laboratory based on the Agilent MassHunter Personal Compound Database and Library software and information from the LIPID MAPS Lipidomics Gateway. Using this customized sphingolipid database and the “find-by-formula” option in the MassHunter Qualitative Analysis Software (version B.06.00), potential sphingolipids were identified based on a comparison of accurate mass, abundance of the isotopes, and isotope spacing with the calculated theoretical masses and abundances. The structures of potential identified sphingolipids were further determined according to the characterized fragments obtained from high-resolution MS/MS data.

The raw data for quantitative analysis were processed with Agilent MassHunter Quantitative Analysis B.06.00 software. The resulting data were first transferred into a Microsoft Excel-type spreadsheet and then imported into the SIMCA-P^+^ 14.0 software (Umetrics, Umea, Sweden) for multivariate statistical analysis. Principal component analysis (PCA) was used to visualize general clustering among the different groups. 3D OPLS-DA was carried out to identify differences in sphingolipid expression between the different groups based on their VIP values. Variables that were significantly changed among different samples were selected as potential biomarkers based on VIP > 1.5 and then validated using t-test analysis.

Analysis of variance (ANOVA) or Kruskal–Wallis tests were performed to examine the differences in the clinical characteristics among the different groups using the SPSS version 21.0 software (SPSS Inc., Chicago, IL, USA). Values are shown as the mean and 95% confidence interval, and a value of *p* < 0.05 was considered statistically significant.

### Ethical approval

All procedures performed in studies involving human participants were in accordance with the ethical standards of the Ethics Committee of the First Affiliated Hospital of Guangzhou Medical University (Reference: 2013039 and 2016064) and with the 1964 Helsinki Declaration and its later amendments or comparable ethical standards.

### Informed consent

Informed consent was obtained from all individual participants included in the study.

## Conclusion

In conclusion, remarkable elevations in the levels of S1P, Cer, and SM were observed in PCOS patients compared to healthy women. Notably, SM (d40:0), SM (d38:0), SM (d18:1/22:0), SM (d18:1/24:0), SM (d41:0) and Cer (d18:0/24:0) showed the most significant alterations, which showed that SM species with long saturated acyl chains were the best candidates to serve as novel biomarkers of PCOS. Our results are in accordance with previous studies suggesting that PCOS is associated with increased SM and that SM species with long saturated acyl chains are closely correlated with the development of metabolic syndrome, obesity, and IR. In all three subgroups of PCOS (NIR, NOIR, OIR), the alteration of sphingolipid levels followed a similar trend as the overall PCOS group except for LacCer, which was only elevated in NIR. Our results suggest that serum sphingolipids might be useful as diagnostic biomarkers for different subgroups of PCOS.
